# Breast cancer incidence and overdiagnosis in Catalonia (Spain)

**DOI:** 10.1186/bcr2620

**Published:** 2010-08-03

**Authors:** Montserrat Martinez-Alonso, Ester Vilaprinyo, Rafael Marcos-Gragera, Montserrat Rue

**Affiliations:** 1 Basic Medical Sciences Department, Biomedical Research Institut of Lleida (IRBLLEIDA), Alcalde Rovira Roure 80, Lleida, 25198, Catalonia, Spain; 2 Basic Medical Sciences Department, University of Lleida, Montserrat Roig 2, Lleida, 25008, Catalonia, Spain; 3 Evaluation and Clinical Epidemiology Department, Hospital del Mar-IMIM, Doctor Aiguader 88, Barcelona, 08003, Catalonia, Spain; 4 Epidemiology Unit, Girona Cancer Registry, Passatge Farinera Teixidor 1, Girona, 17005, Catalonia, Spain; 5 Catalan Cancer Plan, ICO-Hospital Dr. Josep Trueta, Av. França s/n, Girona, 17007, Catalonia, Spain

## Abstract

**Introduction:**

Early detection of breast cancer (BC) with mammography may cause overdiagnosis and overtreatment, detecting tumors which would remain undiagnosed during a lifetime. The aims of this study were: first, to model invasive BC incidence trends in Catalonia (Spain) taking into account reproductive and screening data; and second, to quantify the extent of BC overdiagnosis.

**Methods:**

We modeled the incidence of invasive BC using a Poisson regression model. Explanatory variables were: age at diagnosis and cohort characteristics (completed fertility rate, percentage of women that use mammography at age 50, and year of birth). This model also was used to estimate the background incidence in the absence of screening. We used a probabilistic model to estimate the expected BC incidence if women in the population used mammography as reported in health surveys. The difference between the observed and expected cumulative incidences provided an estimate of overdiagnosis.

**Results:**

Incidence of invasive BC increased, especially in cohorts born from 1940 to 1955. The biggest increase was observed in these cohorts between the ages of 50 to 65 years, where the final BC incidence rates more than doubled the initial ones. Dissemination of mammography was significantly associated with BC incidence and overdiagnosis. Our estimates of overdiagnosis ranged from 0.4% to 46.6%, for women born around 1935 and 1950, respectively.

**Conclusions:**

Our results support the existence of overdiagnosis in Catalonia attributed to mammography usage, and the limited malignant potential of some tumors may play an important role. Women should be better informed about this risk. Research should be oriented towards personalized screening and risk assessment tools.

## Introduction

Breast cancer (BC) incidence rates in women have been increasing steadily in the 1980 s and 1990 s in many countries. Time trends in the incidence of breast cancer have been influenced by different factors: changes in reproductive patterns, the introduction of screening mammography, obesity trends, hormone replacement therapy (HRT), oral contraceptive use and better health awareness [1-10]. In Europe, BC incidence increased in all countries, with or without national screening programs [6]. In Spain, where BC incidence is lower than the European average, incidence rates have increased since 1973, with a more marked rise during the 1990 s [11] in parallel with the dissemination of mammography, both opportunistic and publicly organized screening programs. In Catalonia (Spain), the annual percentage change between 1980 to 1984 and 1995 to 1999 was 2.2% [12] and new cases of female BC were estimated at 4,700 in the year 2008. This quantity represents 30% of all cancer diagnoses in women.

Screening increases incidence rates in three ways [13]. First, there is an immediate rise in incidence, due to the early diagnosis of prevalent asymptomatic cancers [14,15]. Second, age at diagnosis decreases as a result of the lead time introduced by screening (estimated as two to four years) [16]. Third, screening may cause overdiagnosis when it detects tumors which would never have been diagnosed during a lifetime without screening because of the lack of progressive potential or death from other causes [17-21].

Estimates of overdetection of invasive cancer may be affected by important biases. Biesheuvel *et al*. mentioned 1) differences in the underlying breast cancer risk between screened and unscreened populations, 2) contamination of intervention and control groups, 3) screening in the control group after the intervention period ends, and 4) inadequate allowance for lead-time [22]. It seems that the older trial data tend to provide lower estimates of overdiagnosis, whereas the more recent observational data tend toward higher estimates. Since no decline in interval cancer rates has been observed [23] part of the overdiagnosis increase may be related to an increase in the sensitivity of mammography (both for cancers that will progress and for tumors of limited malignant potential).

Mathematical modeling may overcome some of the previous mentioned biases. In the US, Holford estimated the contribution of screening to the upward trend in BC incidence using log-linear models for age, period and birth cohort (APC) [24]. Holford's models also provided background estimates of trends that might have been expected as a result of the continuation of historical increases in the rates. The Cancer Intervention and Surveillance Modeling Network (CISNET) used the background estimates as inputs for modeling the impact of screening and adjuvant treatments on BC mortality trends [25,26].

The interest in assessing the impact and cost-effectiveness of breast cancer early detection programs in Catalonia (Spain) led us to work with mathematical models developed by Lee and Zelen within the CISNET [27,28]. The aims of this study are 1) to use reproductive and screening data to model invasive BC incidence trends and to obtain background estimates of invasive BC incidence and 2) to use the Lee and Zelen mathematical models to quantify the extent of overdetection of invasive BC related to screening.

## Materials and methods

### Setting

In Spain there is a National Health System (NHS), financed primarily by taxes, which provides universal and free health coverage, including early detection of breast cancer. Catalonia is an autonomous region of Spain which has approximately one sixth of the Spanish population. The Catalan Breast Cancer Screening Program (BCSP) started gradually, at the beginning of the 1990 s, providing biennial mammography screening tests to women 50 to 64 years old. Since the year 2000, women older than 64 are kept in the program until the age of 69. Before the start of and in addition to the BCSP, there has also been a certain degree of opportunistic breast cancer screening done in the public and private health care sectors. In the 1994 Catalan Health Survey, when most of the screening in Catalonia was opportunistic, rates of screening mammography were 43% in women aged 40 to 49 years and 27% in women aged 50 to 64 years [29]. In 2004, 61.2% of the invited women participated in the BCSP and 75.7% either participated in the BCSP or reported that they had received recent mammograms (non-published BCSP data).

### Data

To model invasive breast cancer incidence rates in Catalonia, age and period specific incidence data were obtained from the Girona and Tarragona population-based cancer registries in Catalonia (both provinces representing 18.5% of the total Catalan population and covering either urban and rural areas). The Girona and Tarragona provinces have around 750,000 and 800,000 inhabitants, respectively. Data from Girona was provided directly by the Girona Cancer Registry and data from Tarragona was downloaded from the International Agency for Research on Cancer registries (IARC) [11]. Incidence data were available for calendar years 1980 to 1989 and 1991 to 2004 for Girona and 1983 to 1997 for Tarragona. Given that the breast cancer incidence rates in the Girona and Tarragona registries are similar [30], both data sources were combined. Numerators for the incidence rates were calculated adding the number of incident cases from both registries by age and calendar year. Denominators were calculated adding the number of women at mid-calendar year, in the Girona and Tarragona provinces, and were obtained from official census population data [31]. We have assumed that the estimated incidence rates were representative of the breast cancer incidence in Catalonia. Ductal carcinoma in situ (DCIS) has not been included in the analysis.

The research protocol was approved by the institutional review board and ethics committee of the Hospital Universitari de Bellvitge (Barcelona) which waived the need for informed consent.

### Breast cancer incidence models

We modeled the **observed incidence **of invasive BC for Catalan women aged 25 to 84 during the time period 1980 to 2004 using an age-cohort model that incorporated cohort characteristics like intensity of mammography utilization and fecundity rate. We did not introduce a period effect because screening mammography was disseminated gradually in our country. We used this model to estimate the **background incidence **of BC under the assumption of no screening. Then, using a probabilistic model that takes into account background incidence, competing risks, distribution of sojourn time in preclinical state, sensitivity of mammography and the dissemination of screening in Catalonia, we estimated the increased age-specific incidence due to lead time (**expected incidence**). Finally, we estimated **overdiagnosis **comparing the observed and expected cumulative incidences, by cohort of birth. In the following sections there are the models' details.

#### Observed incidence model

Observed incidence rates were fitted using an age-cohort model where the cohort effects were split into three components. The first component refers to the fecundity of the cohort and was measured using the cohorts' completed fertility rate (CFR). CFR is the average number of births per woman up to the end of the childbearing years [32]. There is evidence that high parity is protective of breast cancer independent of ages at first and last full term pregnancies [33]. The second component refers to the intensity that mammography was used for each specific cohort and was measured as the proportion of women who were having periodic mammograms for early detection at age 50 (PM50). This value was obtained from a previous work of modeling mammography dissemination in Catalonia (see the details in Appendix A in Additional file 1) [34]. Finally, the third component refers to the remaining cohort characteristics once fecundity and mammography use have been taken into account and for which we do not have data over time, like use of HRT, use of oral contraceptives, obesity, diet or health awareness. The third component has been included in the model as the year of birth of the cohorts.

Our BC incidence model uses age, CFR, PM50 and year of birth to estimate the number of BC incident cases by age and cohort of birth. It assumes a Poisson distribution of the incident cases and takes into account the exposed population. We used fractional polynomials to describe the age and cohort effects in order to increase the exibility of conventional polynomial models and avoid undesirable artifacts of high-order curves (see Appendix B in Additional file 1 and reference [35] for more detail). Since mammography use (PM50) and CFR had opposite trends in most of the studied periods, and PM50 was strongly associated with BC incidence, we decided to include CFR in the model as an *offset *with the coefficient -0.15. This value was obtained from the literature and indicates a relative risk of BC equal to 0.85 for each child born [1,2,36].

Goodness of fit was assessed using the deviance and the likelihood ratio test with respect to the saturated model. Overdispersion of the Poisson model was assessed. Plots of residuals versus fitted values and predictors were assessed to check for lack of fit related to the scale of predictors (data not shown). Confidence intervals of the predicted values were obtained using the delta method and bootstrap.

#### Background incidence model

BC incidence in absence of screening, by cohort of birth, was derived from the BC observed incidence model by considering that the proportion of women having periodic mammograms at age 50 (PM50) was zero. Confidence intervals for background incidence were obtained using the delta method and bootstrap.

#### Expected incidence and overdiagnosis estimation

**Figure 1:**
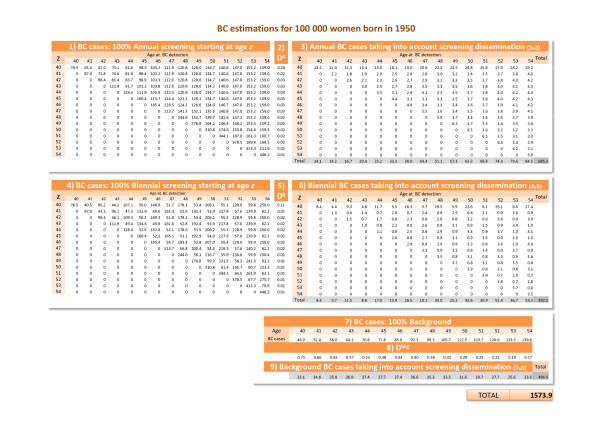
**Steps for estimating overdiagnosis for cohort 1950**. See Appendix E.1 in Additional file 1 for a detailed description.

Using the probabilistic model developed by Lee and Zelen (LZ) for the CISNET [28] we estimated expected BC incidence if women in the population had used mammography as they reported in health surveys, and overdiagnosis was zero. This estimate takes into account the lead time that results when breast cancer is diagnosed earlier. Then the difference between the observed and expected incidence provided an estimate of overdiagnosis. The steps were the following (Figure 1):

1. We considered all the scenarios that assumed a) 100% of women starting screening mammography at age *z *with 40 ≤ *z *≤ *w*_max_, where *w*_max _is the highest age attained by each cohort and b) the periodicity of the exams was annual or biennial. For instance, for cohort 1950, *w*_max _was 54, then *z *took 15 different values. Therefore, for this cohort 30 different scenarios were computed (15 with annual screening and 15 with biennial screening).

2. We used the LZ model to estimate the number of breast cancer incident cases by age and cohort of birth in each of the scenarios mentioned in step 1, assuming that each cohort had 100,000 women at birth. See the equations in Appendices C and D in Additional file 1 and [28,37-40] for more detail.

3. We considered the dissemination of mammography use in Catalonia by age and birth cohort in order to obtain the scenario that best represents the real pattern of mammography use for each cohort. Weighting the estimates obtained for each scenario in step two by the pattern of mammography use, we obtained the estimated number of incident cases by age and birth cohort (see Appendix B in Additional file 1).

4. For each cohort we obtained the expected cumulative incidence (per 100,000 women at birth) adding the incident cases obtained in step three. We represent this estimate by *CI _e_*.

5. We estimated the observed cumulative incidence by 100,000 women at birth (*CI _o_*) multiplying the observed age-specific incidence rates by the probability of being alive at each age, and adding up all these values.

Finally the estimates of overdiagnosis by cohort of birth were obtained using the formula:

$$100\times \frac{CI_{o}-CI_{e}}{CI_{e}}$$

Appendix E in Additional file 1 shows the calculation of overdiagnosis for the cohort born in 1950 as an illustrative example.

The steps taken to obtain confidence intervals of the overdiagnosis estimates are described in Appendix E.2 in Additional file 1.

#### Sensitivity of the overdiagnosis estimates to changes in relevant parameters

We obtained new estimates of overdiagnosis by modifying the following parameters:

1. Mean sojourn time in pre-clinical state (*α*). In the LZ model *α *takes values in the range of two to four years, depending on age. We estimated overdiagnosis when *α *= 1 and when *α *= 5 for all ages. These scenarios would represent tumors growing faster or slower than in the LZ model, respectively.

2. Mammography sensitivity (*β*). In the LZ model *β *varies from 0.35 to 0.8, depending on age of the woman and year when mammography was performed. We estimated the overdiagnosis assuming *β *= 0.9 for all ages.

3. Repeat mammography behavior. Since the distributions of periodicity of mammograms in Catalonia were quite stable along different ages and calendar years, we obtained new estimates of overdiagnosis in the most extreme situations:

a. 1994 Health Survey for the age-group 40 to 49 years (annual = 0.76, biennial = 0.21, irregular = 0.04).

b. 2006 Health Survey for ages from 60 to 69 years (annual = 0.52, biennial = 0.35, irregular = 0.13).

### Software

The Stata SE/10 statistical package was used to fit the Poisson model for BC incidence, to bootstrap the residuals and to obtain confidence intervals [41]. The Grid Mathematica v6 program was used to apply the stochastic LZ model, to estimate the number of BC incident cases under different screening scenarios, and to estimate overdiagnosis [42].

## Results

**Figure 2:**
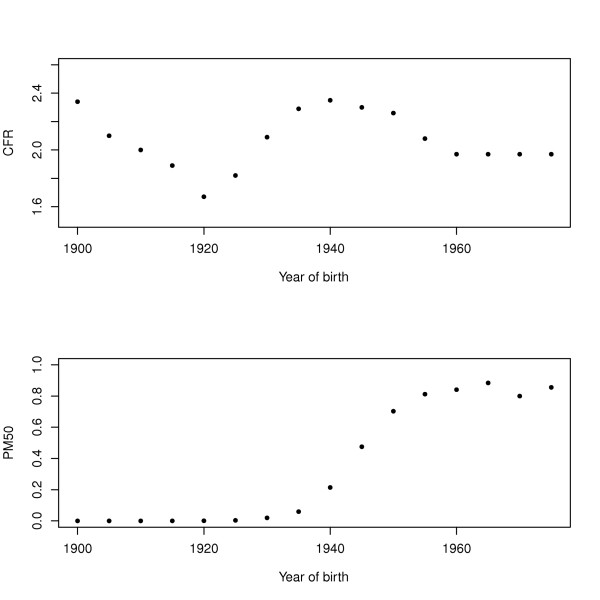
**Completed fertility rate (CFR) and proportion having periodic mammograms at age 50 (PM50)**.

Figure 2 shows the trend over time of the completed fertility rate (CFR) (2a) and of the proportion of women receiving periodic mammograms at age 50 (PM50) (2b), two of the cohort variables used to model BC incidence. Figure 2a shows that CFR decreased for women born between 1900 and 1920 and then increased from 1920 to 1940, reaching a maximum of 2.35 children for women born in 1940. After, the CFR decreased again. Figure 2b shows a big increase in mammography usage from 1935 to 1955. For a woman born after 1955 the values of PM50 stabilized at approximately 0.8. It is important to note that for women born between 1940 and 1955 we observed both a decrease of CFR and an increase of PM50.

Table 1 shows the equation, the estimated coefficients and standard errors of the BC incidence model. Fractional polynomials selected powers two and three for age, lineal for year of birth and power 0.5 for PM50. Independent variables were centered with respect their means. The model coefficients indicate that incidence increased with age, year of birth and exposure to periodic mammography. The square root effect of PM50 on BC incidence indicates that the impact of mammography use in BC incidence attenuated as PM50 increased. The effect of fecundity on BC incidence was considered inverse and constant overtime.

**Table 1:** Breast cancer incidence model; Catalonia 1980 to 2004

	**Coef**.	Std. error	*P***-value**
Age_1_	-38.8418	1.0854	< 0.001
Age_2_	0.0005	0.0002	0.002
PM50_1_	0.6250	0.9878	< 0.001
YB_1_	0.0111	0.0026	< 0.001
Constant	-6.0626	0.0156	< 0.001

The dependent variable is the number of observed incidence cases. The model has an offset, which is a term with coefficient constrained to 1. offset = log(exposed) 0.15 CFR, Age_1 _= (age/10)^-2 ^- 0.0331, Age_2 _= (age/10)^3 ^- 166.375, PM50_1 _= PM50^0.5 ^- 0.4342, YB_1 _= year-of-birth-1937.5. CFR: completed fertility rate, PM50: proportion of women who were having periodic mammograms for early detection at age 50. Deviance = 482.53. The expected number of incident cases, *E*(*I*), can be obtained using the equation: *E*(*I*) = exp(-6.0626 - 38.8418 Age_1 _+ 0.0005 Age_2 _+ 0.6250PM50_1 _+ 0.0111YB_1 _+ offset).

**Figure 3:**
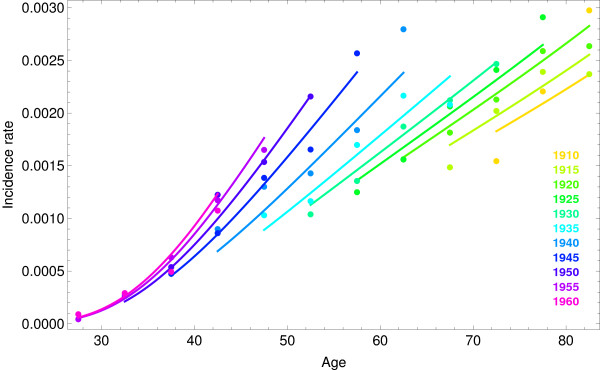
**Observed breast cancer (BC) incidence rates per 100,000 women (points) and the fitted model (lines)**. Each color represents a cohort of birth.

The observed BC incidence rates and the estimation provided by the model can be observed in Figure 3 grouped by year of birth. There was agreement between observed and predicted values. Data shows an increase in BC incidence, especially in cohorts born from 1940 to 1955. The biggest increase of BC incidence was observed for ages 50 to 65 years, where the lowest BC incidence rate was less than half of the highest. The slopes of the estimated rates for the oldest cohorts were nearly parallel but we observed increasingly steeper slopes for the 1935 and younger cohorts.

**Figure 4:**
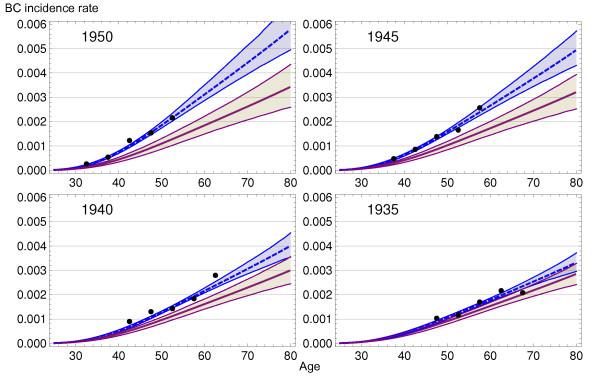
**Breast cancer (BC) incidence model for *screening *and *background *scenarios**. Each plot shows the results for cohorts born in 1935, 1940, 1945 and 1950: observed BC incident rates per 100,000 women (points), model with (dashed blue line) and without (purple line) screening. Confidence intervals were obtained using bootstrap.

Figure 4 shows the observed (dots), estimated (blue dashed line) and background incidence rates for cohorts 1935, 1940, 1945 and 1950 (purple line). Confidence intervals correspond to the bootstrap method and show narrow estimations (intervals obtained by the delta method were slightly narrower). Differences between background and screening scenarios were insignificant for woman born in 1935 or before (data not shown) and increasingly higher for woman born later. This provided the first clue to the magnitude of overdiagnosis due to screening.

**Figure 5:**
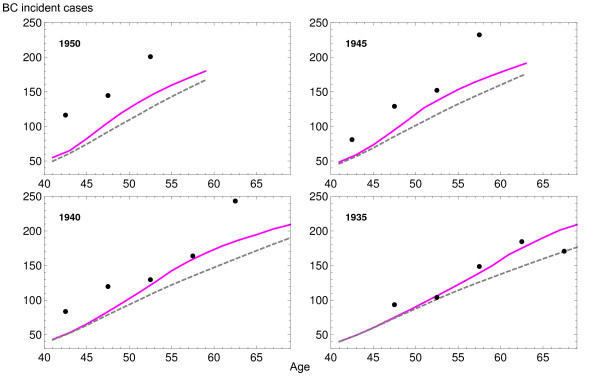
**Predicted breast cancer incidence rates per 100,000 women at birth**. Each plot shows the results for cohorts born in 1935, 1940, 1945 and 1950: observed (points), background scenario (dashed gray line), and scenario that takes into account the actual dissemination of mammography (purple line).

Figure 5 presents the number of incident BC cases that would be obtained from a cohort of 100,000 women at birth in three different situations. The dashed line indicates the number of cases under the background incidence scenario and represents a situation of no mammography use. The solid line indicates the number of cases that would be diagnosed if mammography had been used as reported in the Health Surveys. These estimates were obtained by applying the LZ model to the background incidence. The increase in incidence with respect to the background is due to early diagnosis. Dots represent the number of observed cases for 100,000 women at birth and have been obtained from the observed incidence rates and probabilities of survival. In the absence of overdiagnosis, the dots would appear close to the solid line. The increasing distance between the observed and expected values by cohort of birth indicates that overdiagnosis has augmented over time.

Table 2 shows the overdiagnosis estimates and confidence intervals for cohorts born between 1935 and 1950. These estimates have been obtained by comparing cumulative incidences from 40 years of age to the last observed age in each cohort. Overdiagnosis estimates vary from 0.4%, 95% CI (-8.8%, 12.2% ) for the 1935 cohort to 46.6%, 95% CI (22.7%, 85.2%) for the 1950 cohort.

**Table 2:** Overdiagnosis estimation by year of birth in Catalonia

Cohort	Overdiagnosis (%)	[95% conf. interval]	
1935	0.4	-8.8	12.2
1940	23.3	9.1	43.4
1945	30.6	12.7	57.6
1950	46.6	22.7	85.2

Table 3 shows the results obtained after performing a sensitivity analysis for the overdiagnosis estimates. The largest change in overdiagnosis was observed when modifying mean sojourn time in the pre-clinical state (*α*). A pattern of mammography use caused a small change in overdiagnosis (less than a 5% change between the two extreme scenarios). Finally, the mammography sensitivity caused changes of around 1.5% in the overdiagnosis estimation.

**Table 3:** Sensitivity analysis for the cohort born in 1945

Screening pattern	Parameter change	Overdiagnosis (%)
Annual (*z *= 40)	-	26.4
Annual (*z *= 40)	*β *= 0.9	25.0
Annual (*z *= 40)	*α *= 1	51.1
Annual (*z *= 40)	*α *= 5	18.3
Biennial (*z *= 40)	-	33.9
Mamo dissem 1994 for 40 to 49 years	-	33.8
Mamo dissem 2006 for 60 to 69 years	-	29.3

To test the sensitivity of the model we changed some of the parameters and estimated overdiagnosis for woman born in 1945. The modified parameters were mammography sensitivity (*β*) and mean sojourn time in pre-clinical state (*α*). In the first five scenarios, 100% of the population started receiving mammography at age 40 (*z *= 40). The last two scenarios take into account the actual dissemination of mammography, and use as proportions of repeat mammography behavior the most extreme vañues found in the different health surveys (see Methods).

## Discussion

### Principal findings

Breast cancer incidence in Catalonia has increased during the twentieth century with a more marked rise in cohorts born from 1940 to 1950, who were 30 to 40 years old at the beginning of the 1980 s. The progressive dissemination of mammography in Catalonia was significantly associated with this increase, once age and other cohort characteristics were considered. Our estimates of overdiagnosis ranged from 0.4% for women born in 1935 to 46.6% for women born in 1950.

### Comparison with other studies

#### Incidence trends

Botha *et al*. studied breast cancer incidence and mortality trends in 16 European countries until the mid-1990 s. Increases in incidence occurred in all countries, with or without national screening programs and, according to the authors, were consistent with changes in the risk factors [6]. In the USA, Holford *et al*. also found an increased trend with a peak in the mid-1980 s, when screening began to be more aggressively promoted [24].

In Spain, Pollan *et al*. studied the trend over time of the age-adjusted incidence rate of invasive breast cancer. Incidence increased steadily during the 1980 s and 1990 s and it appeared to decline in 2000 to 2004 in the Spanish provinces were screening had achieved full coverage of the target population in a short time period [43]. In Catalonia, where dissemination of screening took longer, the change in the age adjusted incidence trend was not observed.

In contrast to the previous studies, our incidence model was not only designed to assess the trend of breast cancer incidence over time but to obtain an estimate of the background incidence and overdiagnosis. Our results show a dramatic increase of age-specific incidence rates in cohorts born after 1940 associated to the intensity of mammography use in our region.

#### Overdiagnosis estimates

Overdiagnosis or overdetection estimates range from negligible [15,44] or low [45-48] to moderate [22,49-51] and high (50% or more) [14,52-55]. Some of the studies did not account for lead time bias or for decreases in incidence in older age groups no longer screened [44,46].

A review of the eight randomized trials of mammography found that in recent trials in which the control group was not offered screening, an excess incidence of breast cancer remained after many years of follow-up. In those trials in which the control arm was offered screening, there was no evidence of overdiagnosis, although there was a possible shift from invasive to *in situ *disease [56].

Two systematic reviews intended to shed light on overdiagnosis of breast cancer. One of them, performed by Biesheuvel *et al*. concluded that the most reliable overdetection estimates ranged from -4% to 21% and increased with age [22]. The other systematic review, based on published trends in incidence of breast cancer before and after the introduction of mammography screening [53], included five national screening programs and estimated overdiagnosis in a 35% for invasive cancers.

Two recent observational studies estimated overdiagnosis using population data. Puliti *et al. *[57] evaluated the degree of overdiagnosis of breast cancer 15 years after the introduction of service screening in Florence (Italy). For women aged 60 to 69 years at the start of the screening, the group that had a sufficient follow-up period after the last screening, the authors did not find overdiagnosis. In contrast, Morrell *et al. *[58], in New South Walles (Australia) reported overdiagnosis estimates of 30% and 42% (depending on the method used) for women 50 to 69 years old. Although the study populations and methods used are different, our results are consistent with both Puliti and Morrell studies. Similarly to Puliti, we obtained no overdiagnosis for cohorts born around 1935, which were in their 60 s when mammography began to be widespread in Catalonia. And, consistently to Morrell, we obtained estimates of overdiagnosis higher than 40% for the younger cohorts that had been intensively exposed to mammograms for early detection.

Zahl *et al*., using a different approach, compared six-year cumulative incidence of invasive breast cancer in a screened and a control group in Norway [51]. All women in the control group were invited to receive a 1-time prevalence screen at the end of the observation period. Since the cumulative incidence among controls never reached that of the screened group (incidence rate ratio = 1.22), the authors suggested that the natural course of some screen-detected invasive breast cancers may be to spontaneously regress. Similarly, Gotzsche *et al*. in the update of the Cochrane systematic review of screening for breast cancer with mammography estimated that screening led to 30% overdiagnosis and overtreatment, a figure consistent with our results [59].

### Strengths and weaknesses

This study has several limitations: 1) Incidence data in Catalonia was not available at the population level. The two Catalan population based cancer registries, at the Girona and Tarragona provinces, cover an area of around 20% of the Catalan population. We have assumed that the incidence of breast cancer from these registries, was generalizable to the Catalan incidence. We think that this assumption is acceptable because even the cancer registries are geographically distant within Catalonia, the incidence estimates were close.

Besides, both cancer registries report to the International Agency for Research on Cancer and comply with its quality control procedures. 2) Incidence estimates were available for a 25-year period, therefore some age-specific incidence rates were not available. Although the incidence model estimates were stable and precise, the estimates of overdiagnosis for some cohorts had wide confidence intervals. 3) We did not have information on trends over time of important risk factors like HRT, oral contraceptives, alcohol consumption obesity and sedentarism. We know that the use of HRT has been low in Spain. During the 1990 s, the prevalence of HRT use among Spanish women aged 45 to 64 increased progressively reaching a value of 5.9% in 1998 and declined to 4.2% in 2006 [60,61]. Within a cohort of participants in a population-based breast cancer screening program in the city of Barcelona, the prevalence HRT peaked in 2002 at 11% in 50- to 54-year-olds and at 10.1% in 55- to 59-year-olds, followed by a sudden reversal and a progressive decrease [62]. Prevalence of overweight and obesity in Spain has increased as in the majority of other developed countries. A study of primary care users in the Girona province showed that the proportion of women with obesity (*BMI *< 30 kg/m^2^), in the 35 to 44 age group, increased from 6.9% in 1986 to 1989 to 12.9% in 1995 to 1999 [63]. The scarcity of information on risk factors other than fecundity and mammography use led us to include the *year of birth *in the model to represent the remaining cohort effect. 4) Our breast cancer incidence model used grouped data to estimate the association between incidence rates and characteristics of the exposed population at different periods of time. Grouped data analysis may be affected by the ecological fallacy or failure of aggregate level associations to properly reflect individual level associations. We intended to overcome this problem forcing the *completed fertility rate *variable to be inversely associated with breast cancer incidence as reported in the literature. In addition, we included the effect of *year of birth *in the incidence model as linear consistent with an extended increasing trend over time. This assumption provided a more conservative estimate of overdiagnosis than when we fitted a higher degree polynomial function. 5) The estimates of mean sojourn time in a preclinical state that we have used are based on data from the early detection randomized clinical trials [64] which did not take overdiagnosis into account. That would have caused mean sojourn time to appear longer than it was [54]. If mean sojourn time in a preclinical state was smaller than the values we used, our estimates of overdiagnosis would be conservative (see Table 3 where our sensitivity analysis shows the effect of changes in mean sojourn time).

The principal strength of our study is the use of probabilistic models to obtain the expected incidence of breast cancer. Based on the background incidence and the dissemination and patterns of use of mammography in Catalonia we have estimated the increased age-specific incidence due to lead time. Our study does not compare a screened group with a control group, it compares the observed incidence rates with the expected ones assuming that screening detects earlier invasive tumors that would become apparent later during the womens' life.

In comparison with the conventional age-period-cohort (APC) models, our model includes two specific cohort characteristics, the *intensity of mammography use *and the *completed fertility rate*, which have opposite trends during most of the study period. The agreement between the observed and fitted incidence rates for almost all the studied cohorts indicates the relevance of this variables when explaining incidence changes over time and the difficulties in interpreting APC models when they include only one cohort effect that summarizes divergent information.

## Conclusions

Our results support the existence of overdiagnosis in breast cancer screening by mammography in Catalonia. Since our overdiagnosis estimates were high in cohorts that have not reached the age of 60, where the impact of competing risks is low, it seems that the limited malignant potential of some tumors may play an important role in overdiagnosis. As other authors have recommended [20,21], women should be informed about the benefits and harms of screening and research should be oriented towards assessing individual risk and incorporating it to optimize the effectiveness of screening.

## Abbreviations

APC: age-period-cohort; BC: breast cancer; BCSP: Catalan Breast Cancer Screening Program; CFR: completed fertility rate; CISNET: Cancer Intervention and Surveillance Modeling Network; DCIS: ductal carcinoma in situ; HRT: hormone replacement therapy; LZ: Lee and Zelen; PM50: proportion of women receiving periodic mammograms at age 50; USA: United States of America.

## Competing interests

The authors declare that they have no competing interests.

## Authors' contributions

MM-A developed the age-cohort model, participated in the statistical analysis of results and interpretation, wrote drafts and obtained authors' feedback and participated in the writing of the manuscript. EV developed the computer programs that estimate the effect of screening under different scenarios, provided statistical analysis and interpretation of results and participated in the writing of the manuscript. RM-G provided the incidence data and participated in writing and revising the manuscript. MR co-developed the project that includes this study, performed statistical analysis and participated in the writing of the manuscript.

## Supplementary Material

Additional file 1 **Appendix**. The file contains further details of the model for dissemination of mammography, equations for the estimation of BC incidence, prevalence, mortality, and overdiagnosis. Click here for file
