# Supplementary material for: Breast cancer incidence and overdiagnosis in Catalonia (Spain)
Source: Breast Cancer Res. 2010 Aug 3;12(4):R58. doi: 10.1186/bcr2620 (PMC2949650; doi:10.1186/bcr2620)
Supplement: Additional file 1 — Appendix. The file contains further details of the model for dissemination of mammography, equations for the estimation of BC incidence, prevalence, mortality, and overdiagnosis. [file bcr2620-S1.PDF]

APPENDIX

A Estimation of the proportion of women who were having periodic mammograms for early detection at age 50

The following mixed effects model for dissemination of mammography in Catalonia was estimated in a previous work [28]:

$$p = \frac{\phi_1}{1 + exp[(\phi_2 - age)/\phi_3]} \tag{1}$$

were p indicates the proportion of women receiving periodic mammograms, ϕ_1 ($asym$) is the horizontal asymptote as age increases or the proportion at which the curve levels off, ϕ_2 ($xmid$) indicates the age value at which approximately half of the population is receiving periodic mammograms and ϕ_3 ($scal$) indicates the difference in years between the age at which 3/4 of the population are receiving periodic mammograms and the age ϕ_2.

Dissemination curves were estimated for cohorts born in the calendar period 1938 to 1967. We could not estimate dissemination curves for cohorts born before 1938 or after 1967 because data was scarce. The parameters ϕ_1 and ϕ_3 were estimated as fixed effects and the parameter ϕ_2 as random effect.

As shown in Table A1, for cohorts born in the calendar period 1938 to 1967, the proportions of women having periodic mammograms at age 50 were estimated directly from the dissemination equation, setting $age = 50$. For cohorts born before 1938, we used data from the Catalan Health Survey for year 1994 to obtain the proportion of women having periodic mammograms by cohort of birth. For cohorts born after 1967 we used data from the Catalan Health Survey for year 2006. We used these proportions to estimate the random parameter ϕ_2 for each cohort and then we used the equation (1) to estimate the proportion of women having mammograms at age 50.

Table A1.**Number of women interviewed and percent reporting having periodical mammograms. Catalan Health Surveys, calendar years 1994, 2002, and 2006.**

Birth cohort	Parameter $\phi 2$	PM50	Proportion of women having periodic mammograms
1913-1917	96.92	0.0002	0.0335 [a]
1918-1922	88.99	0.0007	0.0563 [a]
1923-1927	80.24	0.0035	0.1067 [a]
1928-1932	70.93	0.0194	0.2094 [a]
1933-1937	64.69	0.0594	0.2497 [a]
1938-1942	56.78	0.2140 [b]	
1943-1947	50.20	0.4750 [b]	
1948-1952	44.76	0.7025 [b]	
1953-1957	41.12	0.8120 [b]	
1958-1962	39.84	0.8406 [b]	
1963-1967	37.33	0.8840 [b]	
1968-1972	41.61	0.7996	0.2523 [c]
1973-1977	39.08	0.8554	0.1764 [c]

[a]Data obtained from the Catalan health survey 1994. [b]Data estimated from the dissemination model. [c]Data obtained from the Catalan health survey 2006.

B Incidence models

Data for the observed incidence model was grouped in 5-year intervals, using the midpoint of the interval as the representative value. Year of birth was estimated as the incidence period midpoint minus the age midpoint. Since breast cancer incidence is very low before age 25 and it is affected by competing risks after 84 years of age, we modeled incidence data from 25 to 84 years of age.

Fractional polinomials were used to model the age and cohort effects on incidence. A fractional polynomial of degree m with powers $p = (p_1, ..., p_m)$ is defined as:

$$FP(m) = \beta_1 X^{p_1} + \beta_2 X^{p_2} + ... + \beta_m X^{p_m}$$

Powers p are taken from a predefined set, for example $\{-2, -1, -0.5, 0, 0.5, 1, 2, 3\}$ and power 0 means $log(X)$.

Mammography dissemination was incorporated in the model in the following way. A screening scenario is characterized by a) the proportion of women that receive periodic mammographies at each age and by b) the distribution of the exams' periodicity (annual, biennial and higher periodicity or irregular). We used

proportions from the Catalan survey from 2002 for ages in the 50-59 year interval (annual = 0.61, biennial = 0.33, and irregular = 0.06). The proportion of irregular screening was split into two parts and each one of them was added to the proportions of biennial and non-mammography.

The difference between the proportion of women that use periodic mammography at ages $z+1$ and z is an estimate of the proportion of women that started mammography at age z. We assumed that all women that reported having mammograms at age 40 (initial age) had not received mammograms before. Weighting the estimates obtained for each screening scenario by the pattern of mammography use, we obtained the estimated number of incident cases by age and birth cohort.

C Estimation of the probability of surviving free of BC: $S_\nu(t)$

The probability of surviving free of disease, by cohort of birth ν, is one of the inputs of the mathematical model. $S_\nu(t)$ depends on mortality from causes other than breast cancer (competing risks [37-38]) and BC incidence. To estimate $S_\nu(t)$ we performed the following steps:

1. Obtain the central mortality rate at age u from other causes than breast cancer

$$m^{-bc}(u) = m^{\tau}(u) - m^{bc}(u) \tag{2}$$

   The all-cause and BC central death rates are labeled as m^{τ} and m^{bc}, respectively. The numerators of the central rates are the number of deaths in the age interval. The denominators have been approximated by the number in the population midway through the interval:

2. Assume that the instantaneous force of mortality λ_m^{-bc} is constant over the age interval $[u, u+1)$ and can be approximated by $m^{-bc}(u)$.

3. Obtain the incidence central rate $i(u)$ and assume that it can approximate the instantaneous incidence rate λ_i. Breast cancer incidence was estimated according to the incidence model in Table 1.

4. Obtain the hazard of failure either due to being a BC case or dying from other causes (λ^F). We assume that the two hazards are independent.

$$\lambda^F = \lambda_m^{-bc} + \lambda_i \tag{3}$$

5. Solve the differential equation:

$$S'_\nu(t) = \lambda^F S_\nu(t); \quad S_\nu(0) = 1 \tag{4}$$

D Equations for estimating breast cancer incidence probabilities

D.1 Background scenario

This section refers to the *background* scenario where BC is diagnosed only by routine care with no screening. Data obtained under the background scenario will be compared to data obtained under the screening scenario, therefore some of the notation is common for both situations and follows the work of Lee and Zelen [28].

The age at which the study starts is denoted by z. In the screening scenario z is the age at which the first screening exam is performed and $t_0 = 0$ the time at which the first screening exam is performed or origin time.

There are three chronological times (relative to $t_0 = 0$) that must be taken into account in the formulas. These are: $x=$ the time at which the preclinical state, S_p, is entered, (x can be positive or negative), $\tau=$ the time at which the clinical state is entered, and $y=$ the time at death. The corresponding ages are $z + x$, $z + \tau$ and $z + y$ and the sojourn times in S_p and S_c are $(\tau - x)$ and $(y - \tau)$, respectively.

We present the formulas used to estimate BC incidence for a specific cohort ν. Incidence probabilities and mammography sensitivity vary by age and cohort of birth. For simplicity, the index ν that indicates birth cohort is not shown in the notation.

The probability of being incident at the time interval $[t, t + 1)$ can be estimated using the formula:

$$I(t) = \int_{t}^{t+1} S_\nu(u) i(u) du \tag{5}$$

where $i(u)$ is the age and cohort specific incidence at time u.

D.2 Relationship between BC incidence and transition to the pre-clinical and clinical states S_p and S_c

Under screening we do not observe the time at which a BC will become symptomatic. Therefore, the incidence distribution can not be used in the calculations. Instead, the Lee and Zelen models use the transition probability $S_0 \rightarrow S_p$ denoted by $w(t)dt$ and the probability density function of the sojourn time in the preclinical state, $q(t)$ [40].

The following equation shows the equivalence between the estimation of incidence at age $z + k$, $k = 0, 1, 2...$ using the incidence $i(t)$ or the $w(t)$ and $q(t)$ functions.

$$I(z + k) = \int_{k}^{k+1} S_\nu(z + \tau) i(z + \tau) d\tau \approx \int_{k}^{k+1} \int_{0}^{z} S_\nu(\tau - x) w(\tau - x) q(x) dx d\tau \tag{6}$$

Also, the left side of this equivalence was used for the final estimation of *background* BC diagnosis.

D.3 Screening scenarios

This section presents the formulas used to perform the estimations under a generic screening scenario. A pattern of screening is characterized by age interval and periodicity of exams. Survival distribution functions and stage at diagnosis distribution are pattern specific.

Under a screening scenario, Lee and Zelen distinguish whether BC was detected in a screening exam or if it was diagnosed in the interval between two screening exams.

D.3.1 BC detected at exam r

Lee and Zelen considered n examinations given at chronological times $t_0 < t_1 < ... < t_{n-1}$. The first exam is performed at age z and time $t_0 = 0$. Each successive exam r is performed at time t_{r-1}. For instance:

Exam number (r)	1	2	3	...	r	...	n
Exam time for annual screening, in years (t_{r-1})	0	1	2	...	$r-1$	...	n-1
Exam time for biennial screening, in years (t_{r-1})	0	2	4	...	$2(r-1)$	...	2(n-1)

The probability of detecting a BC in the first exam (t_0) is

$$Det(t_0, z) = \beta(z) \int_0^z S_\nu(z-x) w(z-x) Q(x) dx \tag{7}$$

where

$$Q(t) = \int_t^\infty q(x) dx \tag{8}$$

is the tail probability of the sojourn time in the preclinical state.

The probability of detecting a BC at time t_r, $Det(t_r, z)$ takes into account if the transition to S_p was done before ($Det_{pre}(t_r, z)$) or after ($Det_{post}(t_r, z)$) the age z.

$$Det(t_r, z) = Det_{pre}(t_r, z) + Det_{post}(t_r, z) \tag{9}$$

$$Det_{pre}(t_r, z) = \beta(z+t_r) \left(\prod_{i=0}^{r-1} (1 - \beta(z+t_i) \right) \int_0^z S_\nu(z-x) w(z-x) Q(t_r+x) dx \tag{10}$$

$$Det_{post}(t_r, z) = \beta(z+t_r) \sum_{j=1}^{r} \left(\prod_{i=j}^{i<r} (1 - \beta(z+t_i)) \right) \int_{t_{j-1}}^{t_j} S_\nu(z+x) w(z+x) Q(t_r-x) dx \tag{11}$$

D.3.2 BC diagnosed in the interval (t_{r-1}, t_r)

Women diagnosed in the interval (t_{r-1}, t_r) may have entered the preclinical state S_p:

- before age z
- in the interval (t_{r-1}, t_r)
- in the interval (z, t_{r-1})

The probability of being diagnosed in the interval equals to the sum of these three probabilities. We introduced an additional index, s, that allowed to obtain the estimates yearly when the screening scenario has biennial periodicity or higher . Thus, $s = 0$ refers to the first year of the interval and $s = 1$ to the second year and so on, successively.

Transition to preclinical state before age z:

$$Int_{pre}(r, z, s) = \left(\prod_{i=0}^{r-1} (1 - \beta(z + t_i)) \right) \int_{t_{r-1}+s}^{t_{r-1}+s+1} \int_0^z S_\nu(z - x) w(z - x) q(\tau + x) dx d\tau \tag{12}$$

Transition to preclinical stage in the interval (t_{r-1}, t_r):

$$Int_{post1}(r, z, s) = \int_{t_{r-1}+s}^{t_{r-1}+s+1} \int_{t_{r-1}}^{\tau} S_\nu(z + x) w(z + x) q(\tau - x) dx d\tau \tag{13}$$

Transition to preclinical state in the interval (z, t_{r-1}):

$$Int_{post2}(r, z, s) = \sum_{j=1}^{r} \left(\prod_{i=j}^{i<r} (1 - \beta(z + t_i)) \right) \int_{t_{r-1}+s}^{t_{r-1}+s+1} \int_{t_{j-1}}^{t_j} S_\nu(z + x) w(z + x) q(\tau - x) dx d\tau \tag{14}$$

D.3.3 BC diagnosed in an interval after the last exam, $(t_{n-1} + s, t_{n-1} + s + 1)$, $s = 1, 2, ...$

As in previous sections, the probability equals to the sum of the following:

Transition to the preclinical state before age z:

$$Int_{pre}(n, z, s) = \left(\prod_{i=0}^{n-1} (1 - \beta(z + t_i) \right) \int_{t_{n-1}+s}^{t_{n-1}+s+1} \int_0^z S_\nu(z - x) w(z - x) q(\tau + x) dx d\tau \tag{15}$$

Transition to the preclinical state in the interval $(t_{n-1}, t_{n-1} + s + 1)$:

$$Int_{post1}(n, z, s) = \int_{t_{n-1}+s}^{t_{n-1}+s+1} \int_{t_{n-1}}^{\tau} S_\nu(z + x) w(z + x) q(\tau - x) dx d\tau \tag{16}$$

Transition to the preclinical state in the interval (z, t_{n-1}):

$$Int_{post2}(n, z, s) = \sum_{j=1}^{n} \left(\prod_{i=j}^{i<r} (1 - \beta(z + t_i)) \right) \int_{t_{n-1}+s}^{t_{n-1}+s+1} \int_{t_{j-1}}^{t_j} S_\nu(z + x) w(z + x) q(\tau - x) dx d\tau \tag{17}$$

D.3.4 Probability of being diagnosed

The probability of being diagnosed during a specific calendar year is the sum of the probability of being detected at the exam (if it was performed) and the probability of being diagnosed in the interval between two exams or after the last exam.

E Overdiagnosis

E.1 Example

Figure 1 illustrates the estimation of incident BC cases taking into account the pattern of mammography use reported in the Health Surveys and the assumption of no overdiagnosis, for women born in 1950. The highest age observed for the 1950 cohort was 54 years. Table 1) in the figure shows the number of BC cases for 100,000 women at birth if the age at initial mammography use was z. Column $2 - D^A$ contains the proportion of women that started annual screening at age z, Table 3) has been obtained by multiplying Table 1) by column $2 - D^A$ and contains the number of BC cases from women that use mammography with annual periodicity.

Table 4), column $5 - D^B$ and Table 6) in Figure 1 contain the same information for women that use mammography with biennial periodicity and Table 7), column $8 - D^{bkg}$ and Table 9) for women that do not use screening mammography.

Adding the BC cases of Tables 3), 6) and 9), we obtain an expected cumulative incidence of $CI_e = 1573.9$ in the age interval 40-54, per 100,000 women born in 1950.

To obtain the observed BC cumulative incidence per 100,000 women at birth, first we obtain the 5-year age-specific incidence rates from the data (617.7, 768.3 and 1079.3 per 100,000 women in the age groups 40-44, 45-50 and 50-54 years, respectively). Then we multiply these rates by the probabilities of being alive at each age (0.95, 0.94 and 0.93 for the three age-groups, respectively). Adding up these values we obtain an observed cumulative incidence $CI_o = 2307.6$.

Therefore, the estimated overdiagnosis for cohort 1950 in the age interval 40-54 years was:

$$100 \times \frac{CI_o - CI_e}{CI_e} = 100 \times \frac{2307.6 - 1573.9}{1573.9} = 46.6$$

E.2 Confidence intervals of the overdiagnosis estimates

Bootstrapping was used to obtain 95% confidence intervals of the overdiagnosis estimates. We repeated the following sequence 1,000 times:

1. Draw a random sample with replacement of the residuals $Z_i = \frac{O_i - E_i}{\sqrt{E_i}}$ of the Poisson incidence model, with O_i being the observed number of breast cancer cases and E_i the expected number in an age- and cohort-group i.

2. Use the bootstrapped residuals (Z_i^b) to create a new sample of count data using the expression ($O_i^b = max(0, E_i + Z_i^b\sqrt{E_i})$).

3. Use the values O_i^b to fit a new Poisson model for BC incidence and a corresponding background incidence model. When fitting this model we allowed to vary the form of the fractional polynomial for variable age and maintained the same powers for the other variables.

4. Follow the steps described in the *Overdiagnosis estimation* section to obtain a new overdiagnosis estimate.

The 95% confidence interval limits were obtained using the percentiles 2.5 and 97.5 of the overdiagnosis estimates.
